# Mi-RNA-93 and Mi-RNA-152 in the Diagnosis of Type 2 Diabetes and Diabetic Retinopathy

**DOI:** 10.3389/bjbs.2021.10192

**Published:** 2022-01-21

**Authors:** A. A. Saleh, S. M. El-Hefnawy, Z. A. Kasemy, A. A. Alhagaa, M. Z. Nooh, E. S. Arafat

**Affiliations:** ^1^ Department of Medical Biochemistry and Molecular Biology , Faculty of Medicine, Menoufia University, Shebein Alkom, Egypt; ^2^ Department of Public Health and Community Medicine, Faculty of Medicine, Menoufia University, Shebein Alkom, Egypt; ^3^ Department of Ophthalmology, Faculty of Medicine, Menoufia University, Shebein Alkom, Egypt; ^4^ Department of Internal Medicine, Faculty of Medicine, Menoufia University, Shebein Alkom, Egypt

**Keywords:** PDR, T2DM, DM, retinopathy, diabetic retinopathy, microRNA, real-time PCR

## Abstract

**Background and Aim:** Diabetes mellitus (DM) is a chronic disorder with diabetic retinopathy (DR) as one of its main microvascular outcomes, being a prime cause of vision loss. Dysregulation of microRNAs (miRNAs) has been associated with some diabetic microvascular complications such as diabetic retinopathy. This hypothesised changes in the serum of miR-93 and miR-152 in diabetes and diabetic retinopathy.

**Methods:** The study cohort consisted of 80 healthy volunteers, 80 type 2 diabetic patients, and 80 diabetic retinopathy patients, of whom 40 had proliferative (PDR) and 40 non-proliferative retinopathy (NPDR). Serum fasting and 2-hour postprandial glucose (2hPP), glycated haemoglobin (HbA1c), fasting insulin, and HOMA-IR were evaluated by routine methods, miR-93 and miR-152 expression by quantitative real-time PCR.

**Results:** FBG, 2hPP, fasting insulin, HOMA-IR, and miR-152 showed an increasing trend across groups while miR-93 showed a decreasing trend (all *p* < 0.001). Binary logistic regression analysis for prediction of DR found that the most significant were miR-152 (OR 1.37, 95% CI: 1.18–1.58, <0.001), BMI (1.13, [1.07–1.31], *p* = 0.004), duration of disease (1.29 [1.04–1.6] *p* = 0.018), and miR-152 (0.01, [0.0–0.47] *p* = 0.019). The most significant predictors of PDR were miR-152 (OR = 1.47, 95% CI: 1.12–1.92, *p* = 0.005), HOMA-IR (2.66 [1.30–5.45] *p* = 0.007), and miR-93 (0.25 [0.07–0.86] *p* = 0.028).

**Conclusion:** MiR-93 and miR-152 can differentiate patients with diabetes and those with DR. Both miRNAs might be potential biomarkers for diabetes and diabetic retinopathy, and specifically for proliferative diabetic retinopathy.

## Introduction

Diabetes mellitus is a disorder of the endocrine system that is expanding in prevalence worldwide, particularly in developing countries (1). Though it can be managed and its complications reduced with nutritional therapy, physical activity, and drugs, its outcomes still have widespread prevalence (2). The main complications of diabetes are cardiac disease, neuropathy, nephropathy, and ophthalmic complications (i.e., cataracts, retinopathy, and macular edema) (3). Diabetic retinopathy is a prime cause of blindness and affects about 80% of those who have diabetes for 20 years or more (4). The pathogenesis of diabetic retinopathy involves retinal microvascular dysfunction and its clinical features are mainly due to basement membrane thickening, abnormal endothelial proliferation, and angiogenesis (5).

MicroRNAs (miRNAs) are short non-coding single-stranded RNAs, 18–24 nucleotides in length, concerned with moderating gene expression, and reputed to affect the expression of one-third of all genes (6). Dysregulation of miRNAs has been associated with some diabetic microvascular complications as diabetic nephropathy and related to disease progression (7). Also, various miRNAs have been linked with different types of diabetes, as miRNA-223 is linked with the pathogenesis of gestational diabetes (8). Additionally, Kovacs et al. (9), showed that the miRNAs expression profile has changed during diabetic retinopathy. They play an important regulatory role in the process of visual function via involvement in the regulation of the physiological processes such as apoptosis of retinal cells and neovascularization (5).

MiRNA-93 is coded by intron 13 of *MCM7* on chromosome 7 and is metabolically controlled (10). Elevated glucose has reportedly influenced miRNA-93 expression. Additionally, miRNA has been found to control vascular endothelial growth factor (VEGF) level which is associated with the pathogenesis of inflammatory diseases and microvascular diabetic complications (11).

MiR-152 is a member of the miR-148/152 family coded for at 17q21.32 (12). MiR-152 plasma levels are linked to plasma osmolality in diabetes and as such may be involved in the pathophysiology (13). Moreover, pancreatic islets of diabetic patients expressed higher levels of miR-152 and may affect insulin release (14). We hypothesised changes in miR-93 and miR-152 in diabetes and its ocular complications i.e. proliferative and non-proliferative retinopathy.

## Subjects and Methods

This research was fulfilled with the assistance of the Medical Biochemistry department, Endocrinology Unit, at the Internal Medicine department and Ophthalmology Department, Faculty of Medicine, Menoufia University. We tested out the hypothesis in 80 healthy volunteers, 80 patients diagnosed with type 2 diabetes mellitus (T2DM), and 80 diabetic patients complicated with retinopathy. The latter were further subdivided into 40 with proliferative diabetic retinopathy and 40 with non-proliferative diabetic retinopathy. Diagnosis was as per the 2018 Standards of The American Diabetes Association (ADA) (15), these being the presence of any of the following measures: 8-h fasting plasma glucose level of ≥7 mmol/L, a 2-h plasma glucose level of ≥11.1 mmol/L after a 75-g oral glucose tolerance test (OGTT), or a random plasma glucose of ≥11.1 mmol/L, the typical presentation of hyperglycemia (i.e., polyuria, polydipsia, hyperphagia, loss of weight) or hyperglycemic crisis, and a haemoglobin A1c (HbA1c) level of ≥6.5%. The inclusion criterion for diabetic retinopathy group was with different stages of diabetic retinopathy with poor vision (not corrected by refraction). Exclusion criteria were patients with epiretinal membrane and traction at the macula apparent clinically by VOLK (90D), any ocular surgery, media opacity, glaucoma, and any retinal diseases apart from diabetic retinopathy. History taken included the duration of diabetes. Ophthalmic examination was corrected Snellen’s visual acuity, converted to log MAR acuity (Minimal Angle of Resolution) for (statistical analysis, slit lamp examination, fundus biomicroscopy by Volk (90D) and intraocular pressure measurement, clinical examination with anthropometric assessment. Calculation of body mass index (BMI) was completed by dividing body weight expressed in kilograms by height expressed in square meters (16). Investigations include colour fundus photography and Fluorescein angiography (FA). A digital retinal camera system (TOPOCON) was used for FA examination after pupillary dilation with (tropicamide 1%). Regarding FA features, the Degree of diabetic retinopathy was classified according to ETDRS study (17) as follows:—Non-proliferative diabetic retinopathy (Mild: at least one microaneurysm. Moderate: more than just microaneurysms. Severe: haemorrhage and exudates in all four quadrants, venous beading in two or more quadrants, or IRMA in at least one quadrant. Very severe: any patient with two or more of the characteristics of severe non-proliferative diabetic retinopathy).—Proliferative diabetic retinopathy: neovascularization in the retina and or the optic disc, vitreous and or preretinal haemorrhage. Prior to sample collection, written approval agreed by the Human Rights Committee in Research at Menoufia University was obtained from all studied cases and controls.

After 8 h of fasting, 10 ml of venous blood was taken from every subject by sterile vein-puncture for routine insulin, glucose, and HbA1c. Insulin resistance was calculated by the homeostatic model assessment (HOMA) (18). HOMA-IR equals fasting glucose (mg/dl) multiplied by fasting insulin (μIU/ml) then divided by a constant of 405.

Assessment of miR-93 and miR-152 Expression by Real-time PCR: MiRNA was purified from 100 µl of fresh serum samples; total RNA with miRNAs was extracted utilizing a miRNeasy kit (QIAGEN, United States). The quantity and quality of the RNA in our samples were evaluated by NanoDrop instrument (Thermo Scientific, United States). Isolated RNA was kept at −80°C. Furthermore, cDNA was obtained by reverse transcription *via* miScript II RT kit (QIAGEN, United States). The reaction was fulfilled on ice in a total reaction volume of 20 μl, consisting of: 4 μl of miScript HiSpec RT buffer, 2 μl of miScript Nucleics Mix, 2 μl of miScript™ reverse transcriptases, 2 μl of nuclease-free H_2_O, and 10 μl of purified miRNA. Reaction was preceded in a 2720 Applied Bio-systems thermal cycler (Singapore) for one cycle of 37°C for 60 min followed by 95°C for 5 min to inhibit the reverse transcriptase enzyme. The formed cDNA was kept at −20°C until the real-time PCR stage. Real-time PCR was carried out utilizing a miScript SYBR Green PCR kit (QIAGEN, United States). Before reaction processing, cDNA was diluted with nuclease-free H_2_O at a ratio of 1:5, and a net volume of 25 μl was used (12.5 μl of SYBR Green Master Mix, 3.5 μl of nuclease-free water, 4 μl of diluted cDNA, 2.5 μl of miScript universal primer, and 2.5 μl of miScript primer assay). MiRNA-16 was co-amplified for normalization as a reference gene. The following primers were used: mature miRNA-93, CAA​AGU​GCU​GUU​CGU​GCA​GGU​AG; mature miRNA-152, AGG​UUC​UGU​GAU​ACA​CUC​CGA​CU; and mature miRNA-16, UAG​CAG​CAC​GUA​AAU​AUU​GGC​G as a reference gene (miScript primer assay kit, QIAGEN, USA). Samples were analyzed by an ABI 7500 real-time PCR instrument (software V.2.0.1, ABI7500) with cycling settings as: first initiation stage for 15 min at 95°C, then three stages of 40 cycles for 15 s at 94°C, 30 s at 55°C, and 30 s at 70 °C. The expression levels of miRNA-93 and miRNA-152 were standardized to these of miRNA-16 and determined *via* the 2^−ΔΔCt^ method.

Results were analyzed by SPSS version 22 (SPSS Inc., Chicago, IL, United States). Tests of normality were performed. Chi-Squared (χ^2^) and Monte Carlo tests were used for qualitative variables. As the four groups represent a disease spectrum, linear trend analysis using the Jonckheere-Terpstra test was applied to detect whether there was an increasing or decreasing trend across the ordered groups. The Mann-Kendall test was used to detect the presence of linear or non-linear trends [steadily increasing/decreasing or unchanging] in a series of data by estimating the effect size following Jonckheere-Terpstra testing. A Spearman correlation test was used for detecting the strength and direction of association between variables. Binary logistic regression analysis was performed to detect the independent predictors for diabetic retinopathy. Multiple regression analysis using pathway analysis was applied to identify the predictors between our variables. Multiple comparisons were tested using Holm-Bonferroni Sequential Correction. *p*-values are statistically significant after this correction. Sensitivity, specificity, positive and negative predictive values, and receiver operating characteristic (ROC) areas under the curve (AUC) were calculated.

## Results

The four groups were matched for age and sex, and as expected, numerous metabolic and clinical indices increased across the disease (Table 1). It was found miR-93 fell sequentially with the disease spectrum, whilst miR-152 increased. There were significant negative/positive correlations between miR-93 or miR-152 and five major metabolic indices, except fasting insulin in proliferative diabetic retinopathy (Table 2). Sensitivity, specificity, positive and negative predictive values, and ROC AUC curves are shown in Table 3. The highest miR-93 ROC/AUC for predicting different groups was for non-proliferative retinopathy from diabetes, whilst the highest ROC/AUC for mir-152 was in differentiating proliferative retinopathy from diabetes.

**TABLE 1:** Characteristics and laboratory investigations.

	Controls (*n* = 80)	Patients			Trend analysis test	Effect size (95% CI)	*p*-value
		Diabetes mellitus (*n* = 80)	Non-proliferative diabetic retinopathy (*n* = 40)	Proliferative diabetic retinopathy (*n* = 40)			
	Mean ± SD	Mean ± SD	Mean ± SD	Mean ± SD			
Age (y)	57.5 ± 8.6	57.3 ± 9.1	57.3 ± 9.1	56.5 ± 9.4	—	—	0.945
Sex: male/female	48/32	52/28	24/16	20/20	—	—	0.457
Family history of diabetes	—	76 (95%)	36 (90%)	36 (90%)	—	—	0.574
Disease duration (years)	—	4.0 (2.3–8.8)	9.5 (6–16.8)	15.5 (13–17)	7.99	0.50 [0.42–0.58]	<0.001
BMI (kg/m^2^)	22.1 ± 2.6	26.3 ± 2.3	28.0 ± 2.3	28.7 ± 1.9	12.03	0.59 [0.53–0.65]	<0.001
FBG (mmol)	4.8 ± 0.5	11.7 ± 2.8	14.9 ± 4.3	17.9 ± 2.1	15.07	0.74 [0.70–0.77]	<0.001
2hPP (mmol)	4.9 ± 0.5	13.4 ± 3.2	16.9 ± 4.4	19.3 ± 2.3	14.68	0.72 [0.68–0.75]	<0.001
HbA1C (%)	5.3 ± 0.8	9.6 ± 1.1	10.9 ± 1.3	12.1 ± 1.6	14.85	0.73 [0.68–0.77]	<0.001
Fasting insulin	4.1 ± 0.5	21.4 ± 3.1	22.6 ± 4.2	28.1 ± 2.2	14.28	0.71 [0.66–0.76]	<0.001
HOMA.IR	0.9 (0.8–0.9)	10.5 (8.2–13.5)	13.1 (9.9–21.9)	21.7 (21.4–24.5)	14.89	0.73 [0.69–0.77]	<0.001
MiR-93 (fold difference)	1.0 (0.32–1.67)	0.62 (0.41–0.95)	0.19 (0.17–0.31)	0.07 (0.04–0.12)	12.45	-0.61 [-0.69]-[-0.53]	<0.001
MiR-152 (fold difference)	1.0 (0.80–1.63)	5.30 (1.56–9.22)	13.0 (7.7–15.7)	37.1 (18.28–47.50)	14.53	0.71 [0.67–0.76]	<0.001

IQR: interquartile range, Data are expressed as no, %, Mean ± SD or Median [Interquartile range] Chi-square test (χ^2^) or Monte Carlo was applied for qualitative variables. Linear trend analysis using the Jonckheere-Terpstra test was applied to detect whether there was an increasing or decreasing trend across the ordered groups. Effect size was estimated using the Mann-Kendall test to detect the presence of linear or non-linear trends [steadily increasing/decreasing or unchanging] in a series of data following a Jonckheere-Terpstra Test. CI, confidence interval.

**TABLE 2:** Correlation between MicroRNA-93 and MicroRNA-152 and laboratory investigations.

	MicroRNA-93						MicroRNA-152					
	Diabetes mellitus		Non-proliferative diabetic retinopathy		Proliferative diabetic retinopathy		Diabetes mellitus		Non-proliferative diabetic retinopathy		Proliferative diabetic retinopathy	
	*r*_*s*_	*p*	*r*_*s*_	*p*	*r*_*s*_	*p*	*r*_*s*_	*p*	*r*_*s*_	*p*	*r*_*s*_	*p*
Fasting glucose	−0.81	<0.001	−0.66	<0.001	−0.45	0.003	0.89	<0.001	0.71	<0.001	0.61	<0.001
2hPP glucose	−0.68	<0.001	−0.60	<0.001	−0.54	<0.001	0.72	<0.001	0.65	<0.001	0.70	<0.001
HbA1C	−0.64	<0.001	−0.61	<0.001	−0.60	<0.001	0.76	<0.001	0.56	<0.001	0.64	<0.001
Fasting insulin	−0.47	<0.001	−0.65	<0.001	−0.23	0.179	0.67	<0.001	0.72	<0.001	0.23	0.147
HOMA.IR	−0.79	<0.001	−0.68	<0.001	−0.45	0.004	−0.88	<0.001	0.72	<0.001	0.59	<0.001

**TABLE 3:** Sensitivity and specificity of MicroRNA-93 and MicroRNA-152 expression in diagnosis of the studied patients’ groups.

	MicroRNA-93				MicroRNA-152			
	Non-proliferative diabetic retinopathy^a^	Proliferative diabetic retinopathy^a^	Non-proliferative vs. proliferative diabetic retinopathy	Diabetic retinopathy^a^	Non-proliferative diabetic retinopathy^a^	Proliferative diabetic retinopathy^a^	Non-proliferative vs. proliferative diabetic retinopathy	Diabetic retinopathy^a^
AUC	0.99 (0.97–1.0)	0.88 (0.78–0.93)	0.79 (0.69–0.88)	0.92 (0.88–0.97)	0.81 (0.73–0.90)	0.97 (0.91–1.0)	0.88 (0.81–0.96)	0.89 (0.84–0.94)
Cutoff point	≤0.22	≤0.13	≤0.15	≤0.32	≥6.70	≥12.55	≥15.75	≥8.75
Sensitivity%	97%	95%	85%	85%	82%	93%	85%	85%
Specificity%	95%	100%	63%	86%	67%	91%	80%	72%
PPV%	91%	100%	69%	86%	56%	84%	81%	76%
NPV%	99%	98%	81%	85%	88%	96%	84%	83%
Accuracy	96%	98%	74%	86%	72%	92%	82%	79%

a Vs. Diabetes mellitus group.

**FIGURE 1:**
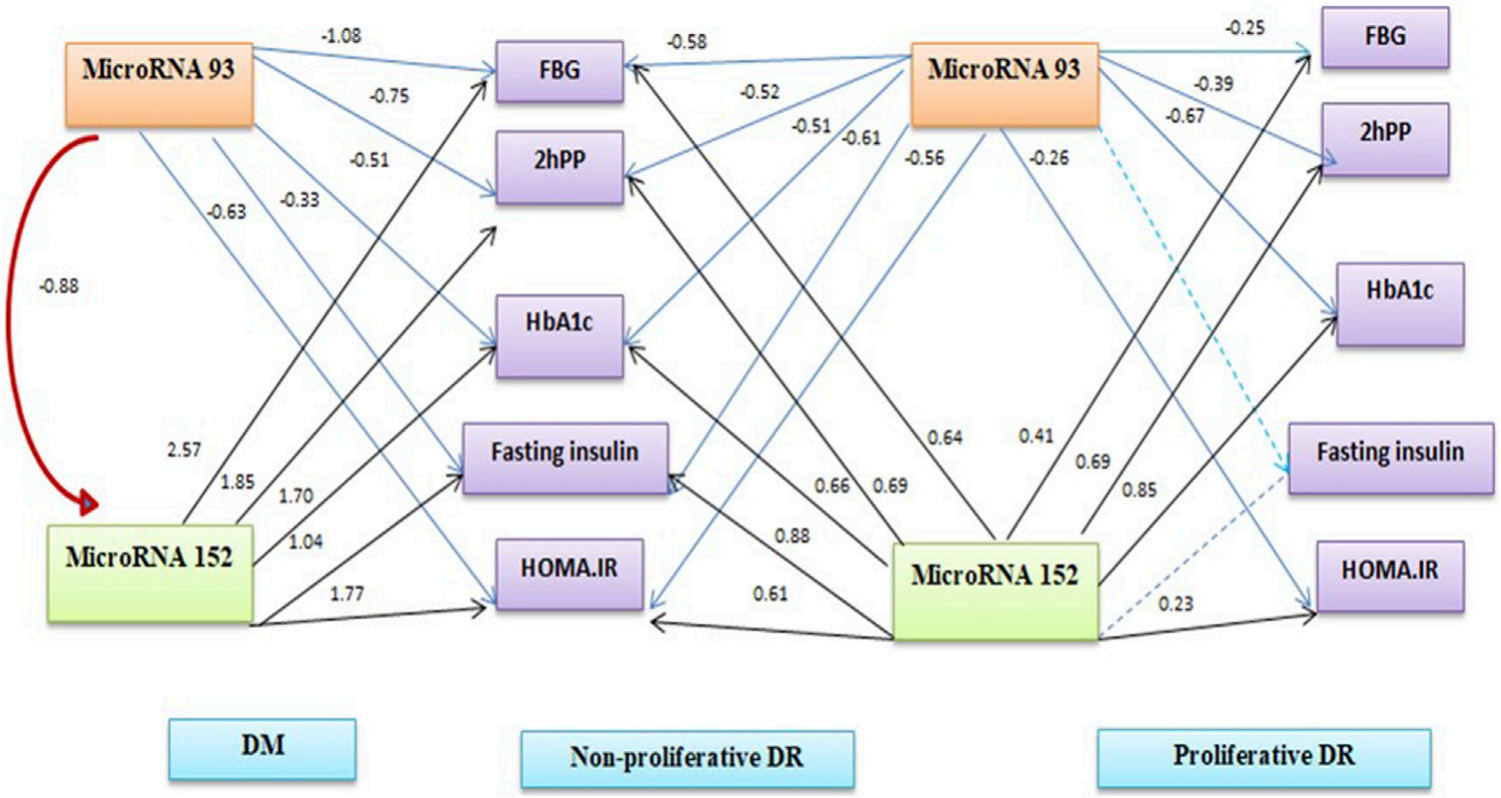
Path analysis diagram of the model used for individual patients’ groups.

Table 4 shows binary logistic regression analyses for the prediction of retinopathy. The most significant predictors of any retinopathy were miR-152 and BMI, for proliferative diabetic retinopathy, the most significant predictors were miRNA-152 and HOMA-IR. Figure 1 summarises the linear regression analysis using a path analysis diagram, showing that miRNA-93 is a significant predictor of fasting and 2-h glucose, fasting insulin, HOMA-IR in all groups, while miRNA-152 is a significant predictor of fasting and 2-h glucose and HOMA-IR in all groups except fasting insulin among the proliferative group.

**TABLE 4:** Logistic regression for Predictors of diabetic retinopathy and proliferative diabetic retinopathy.

	Diabetic retinopathy		Proliferative diabetic retinopathy	
	OR [95% CI]	*p* Value	OR [95% CI]	*p* Value
MiR-152	1.37 [1.18–1.58]	<0.001	1.47 [1.12–1.92]	0.005
BMI	1.49 [1.13–1.96]	0.004	1.39 [0.84–2.31]	0.194
Disease duration	1.29 [1.04–1.60]	0.018	1.08 [0.90–1.29]	0.377
MiR-93	0.01 [0.0–0.47]	0.019	0.25 [0.07–0.86]	0.028
2hPP glucose	0.99 [0.96–1.01]	0.359	0.01 [0.0–3.34]	0.278
HbA1c	1.15 [0.55–2.42]	0.700	0.91 [0.84–1.07]	0.100
HOMA.IR	1.01 [0.83–1.24]	0.861	2.66 [1.30–5.45]	0.007

## Discussion

Diabetic retinopathy is one of the main microvascular complications, and proliferative diabetic retinopathy is the most progressive phase and a serious vision-threatening condition (19, 20). Various studies have investigated roles for miRNAs in a variety of diseases such as diabetic retinopathy (21, 22). We hypothesised differences in the expression of miR-93 and miR-152 in type 2 diabetes and one of its main complications; diabetic retinopathy. Our results revealed a trend to decrease levels of miR-93 across patients with diabetes, non-proliferative diabetic retinopathy, then proliferative diabetic retinopathy compared to controls, while the expression level of miR-152 showed a gradual increase in these groups. Additionally, both miRNAs were independent predictors of diabetic retinopathy and had good sensitivity and specificity for the diagnosis of diabetes and diabetic retinopathy and its subtypes.

Previously, circulating miR-93 expression was found to be decreased in patients with diabetes versus healthy controls (11, 23). Long et al., (24) in an animal model of type 2 diabetes, showed that hyperglycaemia causes downregulation of miR-93, which our data of decreased miRNA-93 in the diabetic group as compared to controls and its negative correlation with glucose and HbA1C levels in different patients groups supports. Our data adds to that of others who reported overexpression of miR-152 in diabetes with a positive association with HbA1c levels (25), miR-152 upregulation in the islets of a type 2 diabetic model (26), and miR-152 overexpression in type 1 diabetes (27).

Various factors, such as VEGF and transforming growth factor-β (TGFβ) may participate in, and increase the risk of, proliferative retinopathy, and induce epithelial to mesenchymal transition (EMT) (28, 29). Fuchs et al. (30) reported the ability of miRNA-93 to suppress TGFβ-induced VEGFA secretion from retinal pigment epithelium cell lines and to convert TGFβ-induced mesenchymal retinal epithelial cells back to the epithelial-like status, which part-explains our finding of a gradual decrease in miRNA-93 expression across patient groups, with the lowest expression level in patients with proliferative diabetic retinopathy. Similarly, miR-93 expression was reduced in acute ocular hypertension retinae compared to controls and miR-93 upregulation suppressed microglial proliferation, inflammation, and cytokine secretion (31). Moreover, miR-93 was also investigated in diabetic renal vascular complications suggesting its antiangiogenic and antifibrotic properties and showed decreased expression in renal tissue of patients with diabetic nephropathy (32), and in a diabetic kidney model was speculated to affect nucleosome remodeling (10). Others reported an inverse relationship between the expression level of miR-93 and VEGF in patients with endometriosis (33).

MiR-152 has been investigated in other diabetic complications, such as increased expression in diabetic nephropathy, with more marked increases in progressive disease (25). In diabetic foot ulcers, another diabetic complication, miR-152-3p expression was elevated in ulcer tissues as compared to normal foot tissues (34). The authors showed that miR-152 targets and decreases the expression of phosphatase and tensin homolog (PTEN) in diabetic foot ulcers. PTEN is identified to control cellular apoptosis and proliferation (34). This relation with PTEN and cellular proliferation was analyzed in another study in nasopharyngeal carcinoma, which revealed that elevated miR-152 expression suppresses apoptosis and enhances invasion and proliferation of malignant cells, which might be *via* downregulation of PTEN (35). These data indicate a relationship between miR-152 and cell proliferation, which might explain our finding of its overexpression in our patients, specifically those with proliferative diabetic retinopathy.

Despite the above, some studies on both miRNAs have provided conflicting results. For example, miR-93 has been reported as up-regulated in diabetic retinopathy (36), and miR-152 downregulation in retinal cells in hyperglycemia (37). Furthermore, the effect of these miRNAs in cancer cell proliferation is also unclear. miRNA-93 was revealed to inhibit malignant cell migration and EMT in breast cancer cells (38), whilst miRNA-93 was overexpressed in glioma cells and related to progressive stages (39). This lack of consensus may be due to the multiplicity of genetic mechanisms involved in cellular proliferation and angiogenesis with the need for more investigations on both blood and tissue samples to clarify any effects.

The current study revealed good diagnostic performances of both miR-93 (downregulation) and miR-152 (upregulation) in diabetes and diabetic retinopathy with a significant correlation with different diabetic biomarkers. We speculate that these miRNAs may be therapeutic targets in the management of diabetic retinopathy. Additionally, both miRNAs are an independent risk factor for diabetic retinopathy. From our results of decreased miR-93 and increased miR-152 across patients with diabetes, non-proliferative diabetic retinopathy then proliferative diabetic retinopathy, we suggest their value as potential biomarkers in diabetes and diabetic retinopathy, specifically, in proliferative diabetic retinopathy. Our data represent an advance in biomedical science in that they show that miR-93 and miR-152 have potential in the assessment and management of the progression of diabetes to retinopathy.

## Summary Table

### What is Known About This Subject?

• Diabetic retinopathy is one of the main microvascular outcomes of diabetes, considered a major source of vision loss.
• Dysregulation of microRNAs (miRNAs) has been associated with some diabetic microvascular complications such as diabetic retinopathy.

### What Does This Study Add?

• MiR-93 and miR-152 can distinguish patients with diabetes from healthy controls, and change in a linear trend with the spectrum of disease severity.
• Both miRNAs might be served as potential biomarkers for diabetes and diabetic retinopathy specifically, proliferative diabetic retinopathy.

## Data Availability

The original contributions presented in the study are included in the article/Supplementary Material, further inquiries can be directed to the corresponding author.
